# Evaluation of patients with dizziness and normal electronystagmography using stabilometry

**DOI:** 10.1016/S1808-8694(15)31327-6

**Published:** 2015-10-20

**Authors:** Adriana Georgia Davim Bastos, Marco Antonio de Melo Tavares de Lima, Liliam Fernandes de Oliveira

**Affiliations:** ^1^ Medical Residence Program, Master studies under course; ^2^ Ph.D., Professor, Coordinator of the Post-graduation course in Otorhinolaryngology, UFRJ; ^3^ Ph.D. Professor, Head of the Department of Physical Activity Biosciences, EEFD/UFRJ

**Keywords:** stabilometry, dizziness, diagnosis, electronystagmography, posture, vestibular function tests

## Abstract

**T**he causes of dizziness are difficult to be diagnosed. At present we have a variety of tests and exams but none of them can adequately evaluate the vestibular function. The most commonly used tests are electronystagmography and posturography. **Aim**: The objective of this study was to analyze the results of stabilometry in patients with complaints of dizziness who had normal results in electronystagmography and to compare them with a control group. **Study Design**: The study was prospective and transversal. **Material and method**: It was conducted at the ENT department, University Hospital, Federal University of Rio de Janeiro. Twenty-two patients (fifteen women and seven men) aged on average 47.6±9 were evaluated. The control group was made up of twenty-five healthy individuals (eighteen women and seven men) aged on average 46.8±7. **Results**: In all analyzed parameters, there were statistically significant differences between the groups. Comparing the results with closed and opened eyes, the anterior-posterior mean velocity in the control group was the only statistically significant result. **Conclusion**: We concluded that the group of patients had statistically significant results in relation to the control group in all the analyzed parameters, showing that the group of patients with complaints of dizziness had more instability in standing position than the group of healthy individuals.

## INTRODUCTION

The maintenance of posture and balance depends on three main systems: visual, vestibular and proprioceptive. Disorders of these complex functions are frequently found in patients with complaints of dizziness, one of the most common symptoms in both otological and neurological clinical practice^1^.

Dizziness is a subjective and nonspecific symptom with varied characteristics. It is caused by different pathophysiological mechanisms, and it may be a common complaint in different diseases. It may be described as a sensation of imbalance, instability, fluctuation, rotation, “empty head”, among others. Vertigo is dizziness with rotation characteristic, originated from the vestibular system^2^.

The causes of dizziness are difficult to diagnose. Currently, there is a wide range of complementary tests (radiological, audiometric, electrophysiological, posturographic, laboratory tests, etc), but none of them can properly assess the vestibular function. The most widely used tests in vestibular assessment are electronystagmographic tests (caloric tests, positional and rotation tests) and posturographic exams.^3^

Electronystagmography (ENG) assessed vestibularocular reflex (VOR) using electrodes that detect spontaneous or induced nystagmus, by lateral ocular movement, positional modifications, caloric and rotation tests. It provides information on symmetry of the vestibular lesion that affects the lateral semicircular canal. Isolated, it does not provide any diagnosis, but it is useful to differentiate central from peripheral causes of dizziness. Owing to the complexity of the vestibular system, tests that assess the vestibulospinal reflex (VSR) have been suggested to support the diagnosis of vestibular diseases, allowing the use of posturographic tests in such situations.^4^

Posturography is a set of techniques that study posture and informs us about the vestibular-spinal function and the compensation reached at this level by any damage to the balance system, regardless of what happens in other levels. The value of studying VSR is similar to that of electronystagmography for the study of VOR and it is an important complement to otoneurological assessment.^5^

Posturography allows quantitative assessment of vestibular-spinal component of body balance. It is performed in static force platforms (stabilometry or statiokinesiometry) and dynamic forces (dynamic posturography). Stabilometry has been used for many authors in clinical practice and research studies. It provides measures of vestibular-spinal function, giving complementary information that is indispensable for the assessment of patients with dizziness, in addition to analyzing sensorial interactions.^6^

Stabilometry assesses posture balance through the quantification of posture oscillations from the orthostatic position in a force platform. It involves monitoring of pressure centers (CP) displacement to the lateral direction (X) and anterior-posterior direction (Y). Normally, the tests are applied under different protocols for the support base (feet together, separated, supported by one foot, etc.), surface (hard or foam) and vision (opened and closed eyes). It has extensive application in the areas of rehabilitation, Otorhinolaryngology, orthopedics, pharmacology, gerontology, sports, etc.^7^

The aspect that motivated the performance of this study was the need to investigate the vestibular-spinal component of body balance in patients with clinical complaint, but with apparently normal otoneurological assessment, in an attempt to find affections that may suggest the presence of any decompensation at this level.

In view of these considerations, the present study intended to analyze the results of stabilometry of patients with complaints of dizziness that presented normal electronystagmography and to compare them to a group of healthy people.

## MATERIAL AND METHOD

The present study was carried out at the Sector of Special Method (SME), Service of Otorhinolaryngology, University Hospital Clementino Fraga Filho (HUCFF), Federal University of Rio de Janeiro (UFRJ).

There were 22 subjects that formed the group of patients (15 women and 7 men) aged on average 47.6±9 years. The control group comprised 25 healthy volunteer subjects (18 women and 7 men) aged on average 46.8±7 years.

It was a prospective sectional transversal study. All subjects were selected among patients in the ambulatory of Otorhinolaryngology, HUCFF/UFRJ, with complaints of dizziness and clinical indication of electronystagmography, to which the results were within the normal range.

The research protocol was analyzed and approved by the medical ethics committee, HUCFF (under number 131/03) complying with the necessary requirements for clinical study with human beings.

All subjects underwent anamnesis and presented normal otoscopy and negative Romberg test when stabilometry was performed. The following subjects were excluded from the study: those that presented clinical history or past history of neurological disease, limiting or disabling musculoskeletal disease, otological or neurosurgical surgery, or if they had been using labyrinthic depressing drugs, benzodiazepinic or anticonvulsant drugs.

They were instructed, in case of use of labyrinthic depressing drug, to interrupt it for at least three days before the test, a period normally used as a routine at SME for the conduction of electronystagmography.

**Figure 1:**
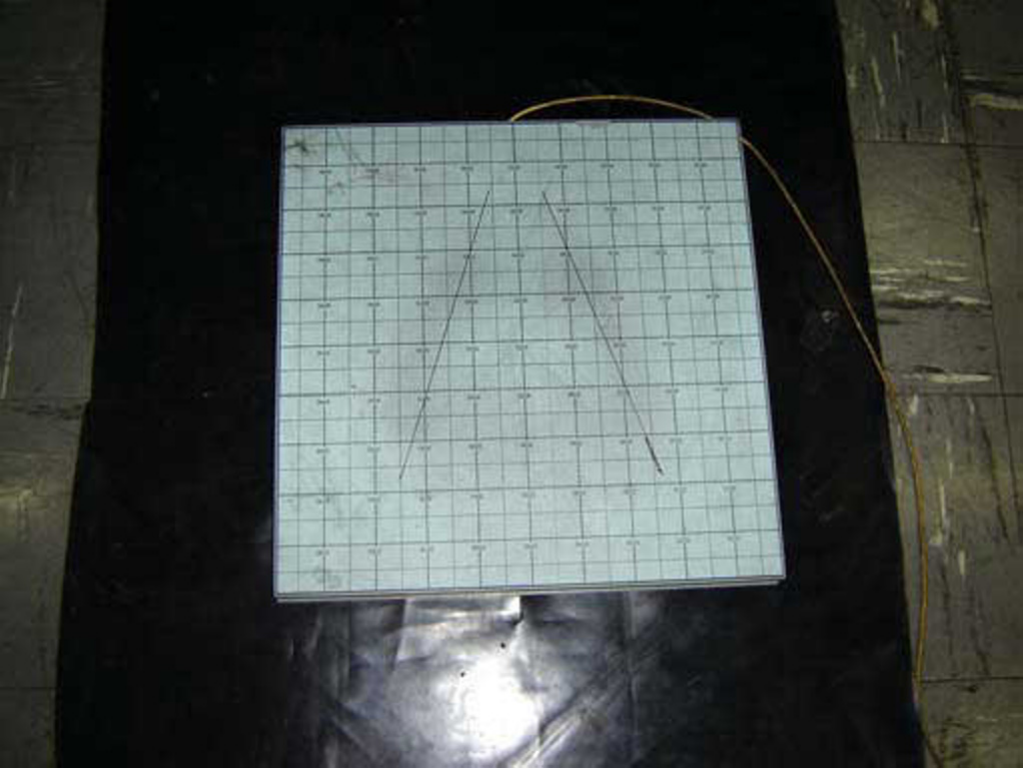
Force Platform AMTI *AccuSway Plus*.

We used the force platform AMTI *AccuSway Plus*, portable, with internal A/D converter of 12 bits and interface RS-232 to communicate with the computer (Figure 1).

**Figure 2:**
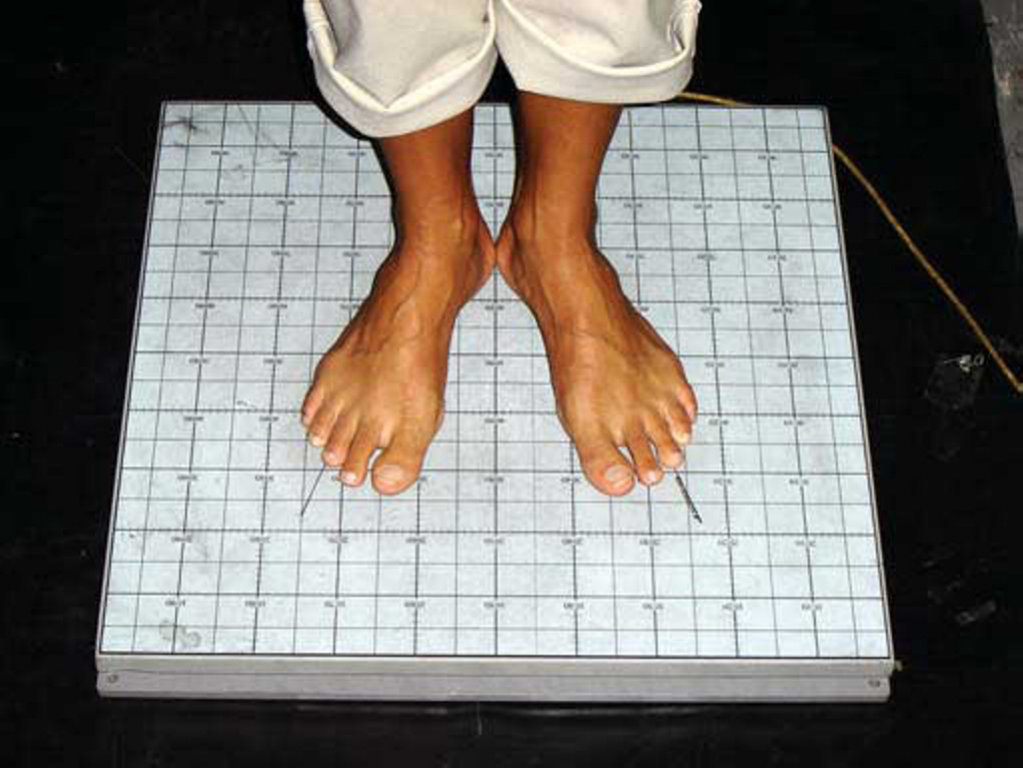
Position of the feet during the test.

**Figure 3:**
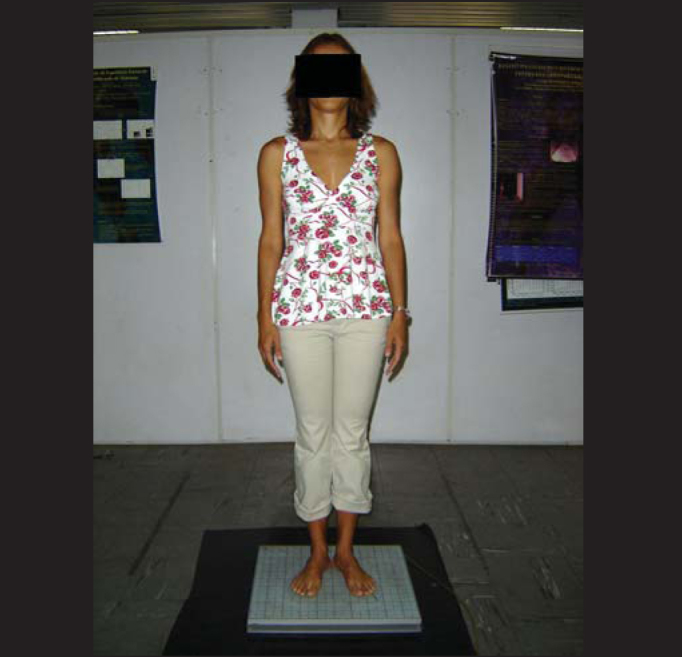
Position of the body during the test.

Exams were performed in a silent environment, with temperatures at about 25º C, in a room dedicated to otoneurological exams. Before the conduction of the exams, the subjects remained seating down at rest for 5 minutes. During the exam, they were asked to take an orthostatic position over the platform, without shoes, feet separated at 30º and heels together, relaxed arms along the body, a position that they should hold for about 1 minute (Figures 2 and 3).

We applied the following tests:

1. opened eyes for 30 seconds and ocular fixation (target at 1.5m);
2. closed eyes for 30 seconds.

**Figure 4:**
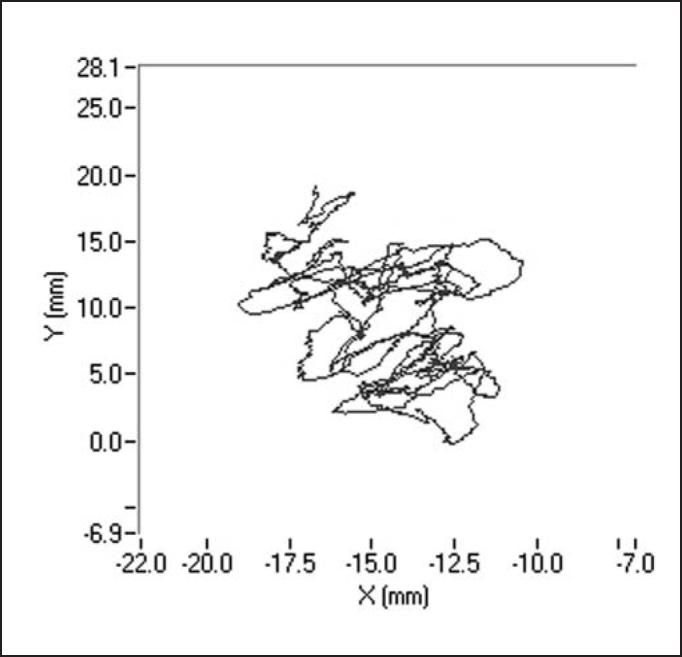
Stabilometric tracing of patient A.M.V with opened eyes.

**Figure 5:**
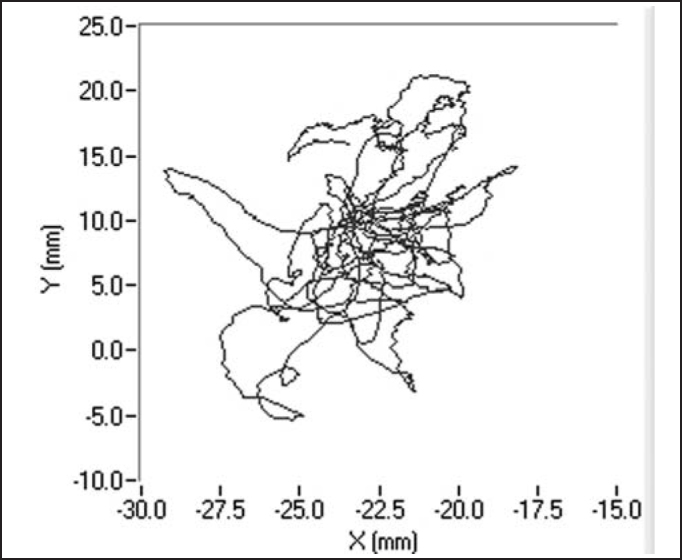
Stabilometric tracing of patient A.M.V with closed eyes.

The recording of signals from the platform was made using three load transducers placed on the surface of the platform and recorded by a microcomputer coupled to the platform, using the software *Balance Clinic* (See the example of a stabilometric tracing with opened and closed eyes in Figures 4 and 5).

To transport and process stabilometric signals, we used a program developed in *Labview 6i (National Instruments)*.

The analyzed parameters were:

1. Average amplitude of pressure center displacement (CP) at lateral plan (AMX).
2. Average amplitude of pressure center displacement (CP) at antero-posterior plan (AMY).
3. Average speed of pressure center displacement (CP) at lateral plan (VMX).
4. Average speed at pressure center displacement (CP) at antero-posterior plan (VMY).
5. Elliptical area of pressure center displacement (CP) at platform plan.

The analysis of data was performed through the software STATÍSTICA 5.1 (stat soft, inc.). To compare the groups, we used t-student test, ANOVA two-way (p = 0.05) test and post-hoc TUKEY HSH test.

## RESULTS

Initially, we made a comparative analysis between the group of patients and the control group, comprising the five studied stabilometric parameters.

**Chart 1:**
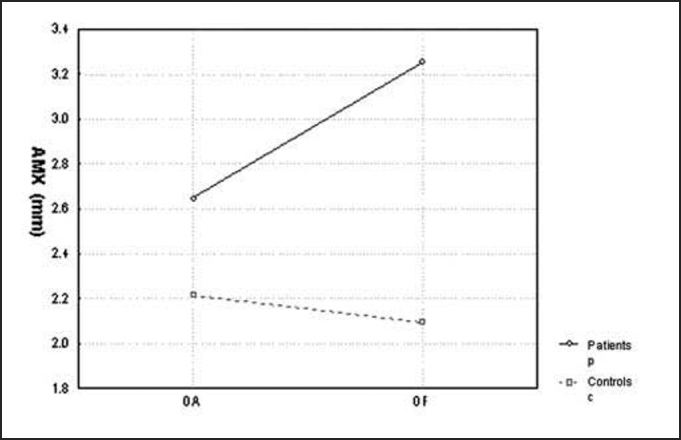
Comparison of mean amplitude of pressure center displacement (CP) at lateral plan (AMX) between the controls and patients with opened eyes (OA) and closed eyes (OF).

**Chart 2:**
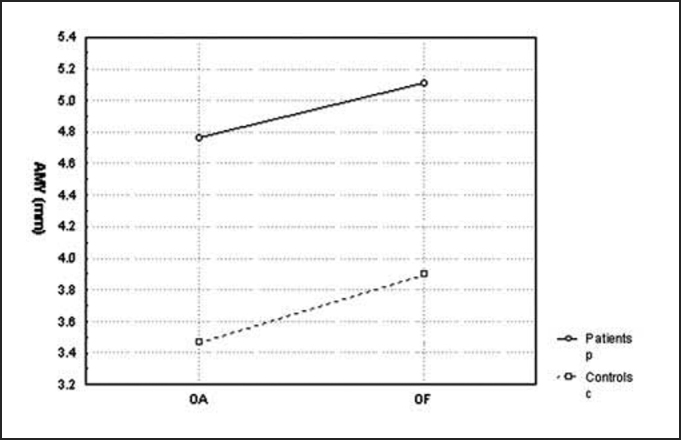
Comparison of mean amplitude of pressure center displacement (CP) at antero-posterior plan (AMY) between the controls and patients with opened eyes (OA) and closed eyes (OF).

Charts 1 and 2 showed average amplitude of pressure center displacement to the lateral plan (AMX) and antero-posterior plan (AMY), which presented statistically significant differences between the groups (p=0.012 and p=0.006, respectively).

**Chart 3:**
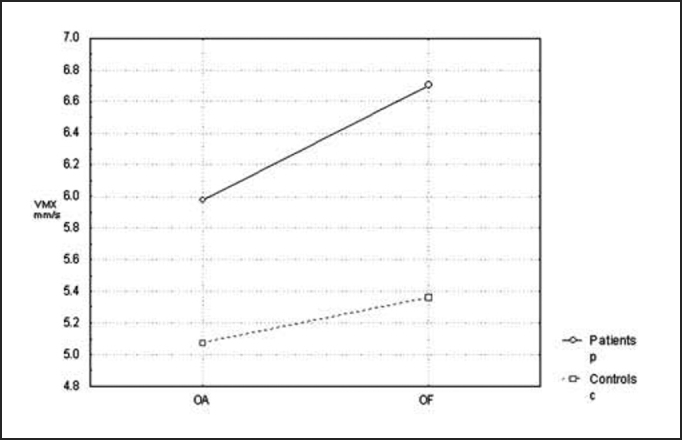
Comparison of mean speed of pressure center displacement (CP) at lateral plan (VMX) between the controls and patients with opened eyes (OA) and closed eyes (OF).

**Chart 4:**
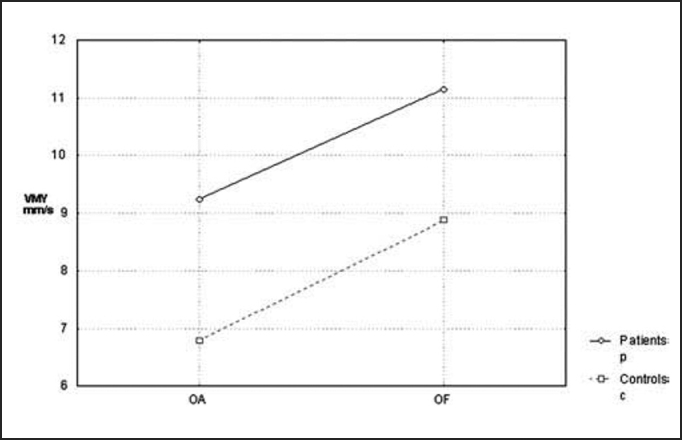
Comparison of mean speed of pressure center displacement (CP) at antero-posterior plan (VMY) between the controls and patients with opened eyes (OA) and closed eyes (OF).

Next, we analyzed the parameter of average speed of pressure center displacement to the lateral plan (VMX) and antero-posterior plan (VMY), which presented compatible results with those in the previous parameters (p=0.047 and p=0.030, respectively), as we can see in Chart 3 and 4.

**Chart 5:**
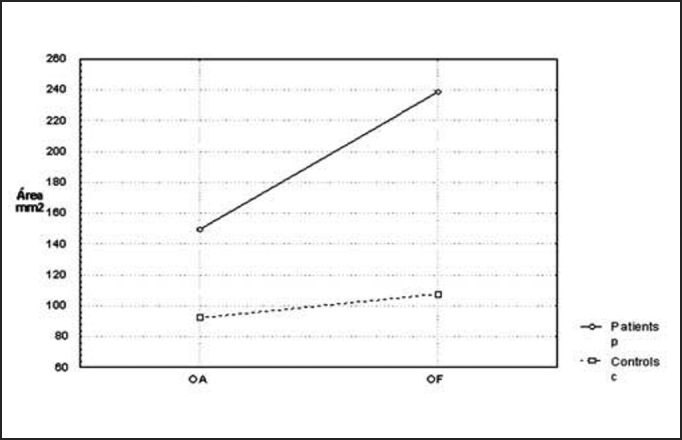
Comparison of mean of elliptical area of pressure center displacement (CP) at the platform plan between the controls and patients with opened eyes (OA) and closed eyes (OF).

The last parameter analyzed, that is, the elliptical pressure center displacement to the platform plan had p value equal to 0.015, and the other stabilometric parameters are shown in Chart 5.

Later, we studied the groups separately, comparing the tests carried out with closed and opened eyes. In the statistical analysis we performed, only average speed of pressure center displacement to the antero-posterior plan (VMY) presented a significant p value equal to 0.008. However, if we compare the remaining stabilometric parameters, these mean values were always higher with closed eyes in relation to opened eyes, in both groups. This tendency can be confirmed by Chart 1, Chart 2, Chart 3, Chart 4, Chart 5.

## DISCUSSION

In the present study, we assessed stabilometric parameters of patients with complaints of dizziness and normal electronystagmography and compared them with the groups of normal subjects. Initially, we showed in Chart 1, Chart 2, Chart 3, Chart 4, Chart 5 a comparative view of the groups. According to what was demonstrated in the Chart profile, the statistical analysis confirmed that groups had a different behavior among each other, and the obtained results were considered abnormal when compared to the control group.

We confirmed that the patients presented instability in the orthostatic position in relation to results in the control group; there are some studies performed with patients with vestibular dysfunction, such as the ones by Gagey 1991; Norré & Forrez 1986; Norrè 1993; Suarez et al. 2000 and Sanz et al. 2004, which were in agreement with ours.2., 6., 8., 9., 10.

Another aspect that we took into account during the assessment was the effect of visual deprivation. It is expected that subjects with vestibular dysfunction have more visual dependency and therefore, upon closing their eyes, they present more affections in their stabilometric parameters.^10^ In our statistical analysis, we observed that only subjects in the control group had statistically significant results and only for the parameter of average speed of pressure center displacement to antero-posterior plan (VMY). However, values of the other studied parameters presented mean values that were higher for closed eyes than for opened eyes. This assumption is visually perceived upon examining the profiles in Chart 1, Chart 2, Chart 3, Chart 4, Chart 5, which confirms the observation of the authors above referred. The absence of other statistically significant results is justified by the small sample size. An increase in sample size could produce a different result, as has been shown by Norré, 1993; Suarez et al. 2000; Sanz et al. 2004 Norré; Black et al., 1978 1993 and Norré 1994, in which they found visual dependency pattern in patients with vestibular dysfunction.8., 9., 10., 11., 12.

Upon assessing vestibular patients in force platforms, especially when we study sensorial interaction, we expect to eliminate visual and/or proprioceptive stimuli, leading to increase in posture oscillations. Trying to confirm this observation Nooré & Forrez (1986) studied 160 vestibular patients using open eyes stabilometry, closed eyes test and head retroflexion. They found abnormal results in half of the assessed patients. To the authors, the results were not significant to support the diagnosis, but they allow functional assessment of the adaptations and compensations that occurred at the vestibular-spinal level, which can be very valuable when we assess treatment and rehabilitation^6^.

Later in 1993, Norré submitted 95 patients with vestibular dysfunction to stabilometric assessment. He assessed area and speed parameters, and observed that in 33 of the patients there was instability in the tests. Next, the author performed vestibular rehabilitation therapy in these unstable patients and when the test was repeated, he observed posture oscillation in most cases confirming, thus, a possibility of using the method to follow up patients undergoing vestibular rehabilitation and opening up new opportunities for study.^8^

Stabilometry, given that it is a quantitative method, can be used repetitively to assess the same patients. Based on such fact, Narita et al. (2004) followed up 31 patients with vestibular dysfunction for a period of approximately 260 days, using measures of serial posture oscillation and the parameters of displacement and area. They found a correlation of clinical improvement and reduction of posture oscillations with time, confirming the usefulness of the test for follow up of patients with vestibular dysfunction.^13^

In summary, the findings demonstrated that the stabilometric parameters chosen are useful to perform functional assessment of patients with complaints of dizziness. They suggest, at least for the results of the present study, that there is a deficient compensation of VSR in relation to the compensation reached for VOR level, considering that the results of electronystagmography were normal. Even though this information is not enough for the diagnosis, it seems to be useful for management, follow up and vestibular rehabilitation, as demonstrated by the reported authors. Thus, many studies are necessary to analyze safety and efficacy of the use of stabilometry in clinical practice.

## CONCLUSION

The group of patients presented statistically significant results in relation to the control group in all analyzed stabilometric parameters, demonstrating that the group of patients with dizziness complaints presented higher instability in the orthostatic position than the group of healthy patients.

## ACKNOWLEDGEMENT

To Mr. José Magalhães de Oliveira, electronic engineer of Laboratory of Biomechanics, School of Physical Education and Sports, Federal University of Rio de Janeiro, for his support in the performance and processing of stabilometric tests.
